# An activated unfolded protein response promotes retinal degeneration and triggers an inflammatory response in the mouse retina

**DOI:** 10.1038/cddis.2014.539

**Published:** 2014-12-18

**Authors:** T Rana, V M Shinde, C R Starr, A A Kruglov, E R Boitet, P Kotla, S Zolotukhin, A K Gross, M S Gorbatyuk

**Affiliations:** 1 Department of Vision Sciences, University of Alabama at Birmingham, AL, USA; 2 Department of Pediatrics, University of Florida, FL, USA

## Abstract

Recent studies on the endoplasmic reticulum stress have shown that the unfolded protein response (UPR) is involved in the pathogenesis of inherited retinal degeneration caused by mutant rhodopsin. However, the main question of whether UPR activation actually triggers retinal degeneration remains to be addressed. Thus, in this study, we created a mouse model for retinal degeneration caused by a persistently activated UPR to assess the physiological and morphological parameters associated with this disease state and to highlight a potential mechanism by which the UPR can promote retinal degeneration. We performed an intraocular injection in C57BL6 mice with a known unfolded protein response (UPR) inducer, tunicamycin (Tn) and examined animals by electroretinography (ERG), spectral domain optical coherence tomography (SD-OCT) and histological analyses. We detected a significant loss of photoreceptor function (over 60%) and retinal structure (35%) 30 days post treatment. Analysis of retinal protein extracts demonstrated a significant upregulation of inflammatory markers including interleukin-1*β* (IL-1*β*), IL-6, tumor necrosis factor-*α* (TNF*-α*), monocyte chemoattractant protein-1 (MCP-1) and IBA1. Similarly, we detected a strong inflammatory response in mice expressing either Ter349Glu or T17M rhodopsin (RHO). These mutant rhodopsin species induce severe retinal degeneration and T17M rhodopsin elicits UPR activation when expressed in mice. RNA and protein analysis revealed a significant upregulation of pro- and anti-inflammatory markers such as IL-1*β*, IL-6, p65 nuclear factor kappa B (NF-kB) and MCP-1, as well as activation of F4/80 and IBA1 microglial markers in both the retinas expressing mutant rhodopsins. We then assessed if the Tn-induced inflammatory marker IL-1*β* was capable of inducing retinal degeneration by injecting C57BL6 mice with a recombinant IL-1*β*. We observed ~19% reduction in ERG a-wave amplitudes and a 29% loss of photoreceptor cells compared with control retinas, suggesting a potential link between pro-inflammatory cytokines and retinal pathophysiological effects. Our work demonstrates that in the context of an established animal model for ocular disease, the persistent activation of the UPR could be responsible for promoting retinal degeneration via the UPR-induced pro-inflammatory cytokine IL-1*β*.

Retinal degeneration encompasses a diverse group of ocular disorders that, despite different pathophysiological mechanisms, are generally characterized by the progressive deterioration of retinal cells, ultimately leading to their death. Although the mechanisms of photoreceptor cell death are still under investigation, recent publications indicate that the UPR^1^ is a common cellular pathway involved in the pathogenesis of age-related macular degeneration, retinitis pigmentosa and diabetic retinopathy. However, the exact role of the UPR in the disease process is unclear.^1^

The UPR, also known as the endoplasmic reticulum (ER) stress response, is a series of evolutionarily conserved signaling pathways aimed at restoring homeostasis under conditions of ER stress.^2^ It is activated in response to the accumulation of misfolded or unfolded proteins in the ER lumen. Operating in a rheostat-like manner, the primary goal of the UPR is to maintain a pro-survival signaling environment. However, if the capacity of the ER to resist stress in the cell is insufficient, the UPR-associated signaling eventually becomes dominant and shifts from a pro-survival to a pro-death cascade. This happens to degenerating the photoreceptor cells regardless of whether the retinal degeneration is the result of genetic defects or prolonged light exposure.^3, 4^

Mice and rats expressing mutant rhodopsin, experience the photoreceptor cell death, much as humans, and manifest the clinical signs of autosomal dominant retinitis pigmentosa (ADRP). For example, the T17M *RHO* mice carrying a human *RHO*, as well as the P23H *Rho* and S334ter *Rho* rats have been used to study the effects of a persistently activated UPR in the retina.^5, 6, 7^ As a result, we have demonstrated not only that the progression of ADRP is associated with an upregulation of UPR markers, but also that ER dysregulation and the onset or progression of retinal degeneration are in fact linked.^8^ Despite these findings, the main question of whether UPR activation is a protective photoreceptor cellular stress response or a factor contributing to retinal pathogenesis in the degenerating retina remains open to debate. Moreover, a mechanism by which the activated UPR could promote retinal degeneration has not yet been proposed. The necessity of understanding the physiological consequences of the UPR in degenerating photoreceptors is obvious, considering UPR activation is often associated with other pre-existing complications in the retina.^9^

Regarding the cell signaling involved in the ER stress-induced retinal degeneration, the links between the UPR and other cellular regulatory processes remain largely unknown. Disruption of ER function broadly impacts other cellular pathways including oxidative stress,^10^ cytosolic Ca^2+^-release^11^ and inflammation.^12^ Thus, all three UPR branches (PERK, IRE1a and ATF6) have been shown to mediate ‘cell autonomous' pro-inflammatory transcriptional programs and contribute substantially to progression of cystic fibrosis, metabolic disorders and intestinal bowel disease.^12^ Therefore, further study of the potential role for the UPR in triggering inflammation during retinal degeneration could give valuable mechanistic insight into retinal pathogenesis. This could in turn help determine if manipulating UPR mediators would be a feasible strategy for fighting inflammation and arresting disease progression in degenerating retinas.

## Results

### A persistently activated UPR promotes loss of photoreceptor function and retinal structure

Tn is known to activate the UPR by inhibiting the *N*-linked glycosylation of newly synthetized proteins resulting in ER protein misfolding and is widely used to experimentally induce the UPR *in vivo* and *in vitro*. We injected Tn into the retinas of ER stress activated indicator (ERAI) mice carrying venus, a variant of green fluorescent protein (GFP) fused with human *XBP1* (X-box binding protein 1) to track UPR activation (Supplementary Figure S1). The results demonstrated that 24 h post injection, the majority of photoreceptors experienced UPR activation. Expression of venus was also observed in other retinal cell types, indicating UPR activation in these cells as well. The impact of UPR activation in photoreceptors was monitored by photoreceptor-derived a-wave amplitudes of the scotopic ERG, SD-OCT-assessed averaged thickness of the outer nuclear layer (ONL) and by performing histological analysis to count the number of photoreceptor nuclei rows.

**Figure 1:**
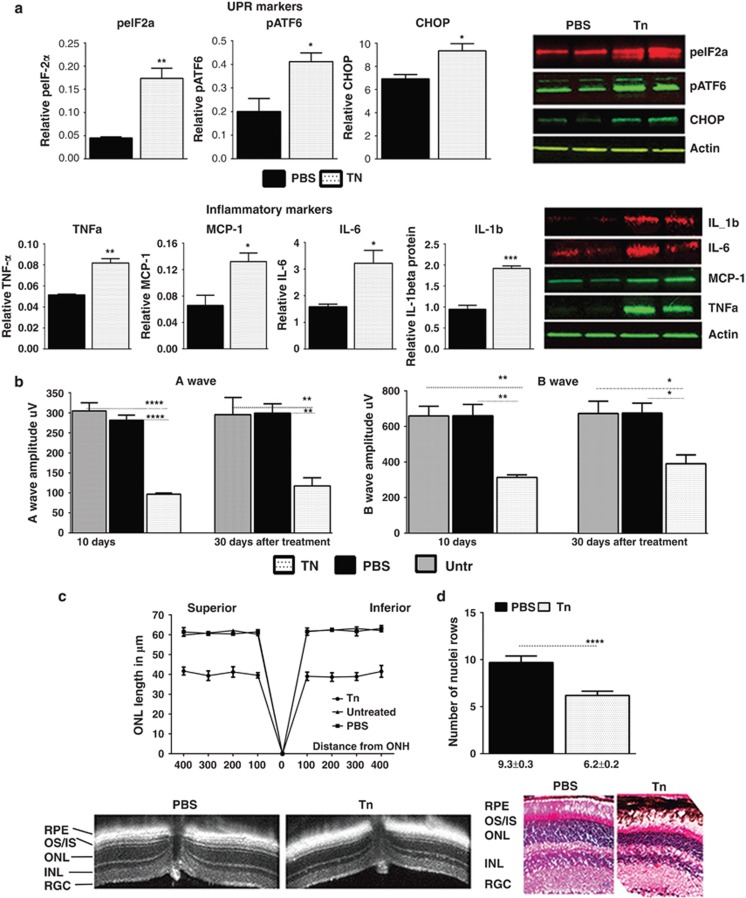
Persistently activated UPR in the wild-type retina induces retinal degeneration. The distribution of data values is shown in S.E.M. (**a**) Western blot analysis of Tn- or PBS-injected retinal protein extracts (*N*=4). Upper: a dose of 0.01 mg Tn activated the UPR in the retina 3 days post treatment. The UPR markers pEIF2a, CHOP and pATF6 were significantly increased compared with PBS-injected retinas (*P*=0.001, *P*=0.028 and *P*=0.020, respectively). Images of western blots are shown on the side. Bottom panel: activation of the UPR was observed concomitantly with the induction of inflammatory signaling in Tn-injected wild-type retinas. The inflammation markers IL-1*β*, IL-6, MCP-1 and TNF*-α* were upregulated 3 days post injection, suggesting that Tn injection induced not only UPR activation but also led to activation of an inflammatory response in the retina (*P*=0.001, *P*=0.029, *P*=0.021 and *P*=0.002). The calculation of the Tn-induced microglial response is shown in Supplementary Figure S1. (**b**) Scotopic ERG responses were significantly reduced in Tn-injected retinas at 10 and 30 days after treatment (*N*=6). Although no difference was observed between the PBS-injected and naive retinas, the a- and b-wave amplitudes were reduced by >60%, 30 days after Tn treatment (*P<0.05, **P<0.01, ***P<0.001 and ****P<0.0001). (**c**) A loss of retinal integrity in Tn-injected wild-type retinas was measured by SD-OCT (*N*=6). A >30% reduction in the ONL thickness was observed across all of the Tn-injected retina compared with PBS-injected mice, ****P<0.0001 at all measurement points. Bottom: SD-OCT images taken from PBS- and Tn-injected retinas. (**d**) Histological analysis following H&E staining of Tn-injected cryostat-sectioned retinas demonstrated a significant loss of photoreceptor cells. The number of rows of photoreceptor nuclei in the Tn-injected retinas was 36% lower compared with PBS-injected mice. Bottom: images of the H&E-stained retinas 30 days after treatment, (*P*=0.001). Scale bar indicates 20 *μ*m

We performed intraocular injection in mice with one of two Tn doses to generate a mild (0.001 *μ*g per eye)^13^ and a strong (0.01 *μ*g per eye)^14, 15^ retinal UPR activation. The contralateral eye was injected with phosphate-buffered saline (PBS) and served as surgery controls. Three days after injection with 0.01 *μ*g of Tn we confirmed the presence of UPR activation markers (Figure 1a and Supplementary Table S1) including the phosphorylated (p) epEIF2*α* (eukaryotic translation initiation factor 2*α*), which was elevated by 3.8-fold. The cleaved form of pATF6 (50 kDa) was also upregulated by 2-fold, suggesting that the Tn-injected retinas were experiencing an ongoing UPR and that the PERK and ATF6 pathways were activated. As a result, we found that the levels of the downstream pro-apoptotic UPR marker CHOP significantly increased by 1.3-fold. The dose of 0.001 *μ*g of Tn per eye failed to produce UPR activation 3 days after treatment.

Next, we analyzed the physiological response of retinas to Tn injection (0.01 *μ*g per eye) by ERG and found a loss of photoreceptor function at 10 and 30 days after treatment (Figure 1b and Supplementary Table S1). The greatest effect was seen in the a-wave amplitude that was decreased by over 60% at 30 days post treatment. In the same retinas, the b-wave amplitudes dropped by 40%. ERG analysis of mice injected with 0.001 *μ*g of Tn demonstrated no alterations in the scotopic ERG a- and b-wave responses at 10 or 30 days post treatment, suggesting that the mild or transient ER stress did not induce retinal degeneration in the wild-type retina.

Then we found that the treatment with Tn resulted in a reduction of the average ONL thickness in the superior and inferior retinas by 35% and 37%, respectively (Figure 1c). Histological analysis of retinal sections confirmed our OCT findings and revealed that Tn-injected retinas lost ~36% of their photoreceptors at 30 days post treatment (Figure 1d).

Altogether, our results demonstrated that Tn-induced UPR activation in photoreceptors promotes progressive retinal degeneration culminating in photoreceptor cell death within the context of the wild-type retina.

### A persistently activated UPR induces an inflammatory response in the wild-type retina

We chose to further investigate if UPR activation had a causal role in the inflammation seen with degenerative ocular diseases (Figure 1a,Supplementary Figure S2 and Supplementary Table S1). Compared with PBS-injected controls, retinal protein extracts from mice injected with 0.01 *μ*g Tn demonstrated UPR activation and showed an upregulation of inflammatory markers TNF*-α*, IL-6 and CCL2/MCP-1 by 1.6-, 2- and 2-fold, respectively. A significant increase in these inflammatory markers suggests a strong inflammatory response. We also found that the microglial response was activated in the Tn-injected retina 3 days post treatment, suggesting a potential cytokine release in response to the activation of ER stress in photoreceptors. A >2-fold increase in IBA1-positive cells was detected in the treated retinas.

**Figure 2:**
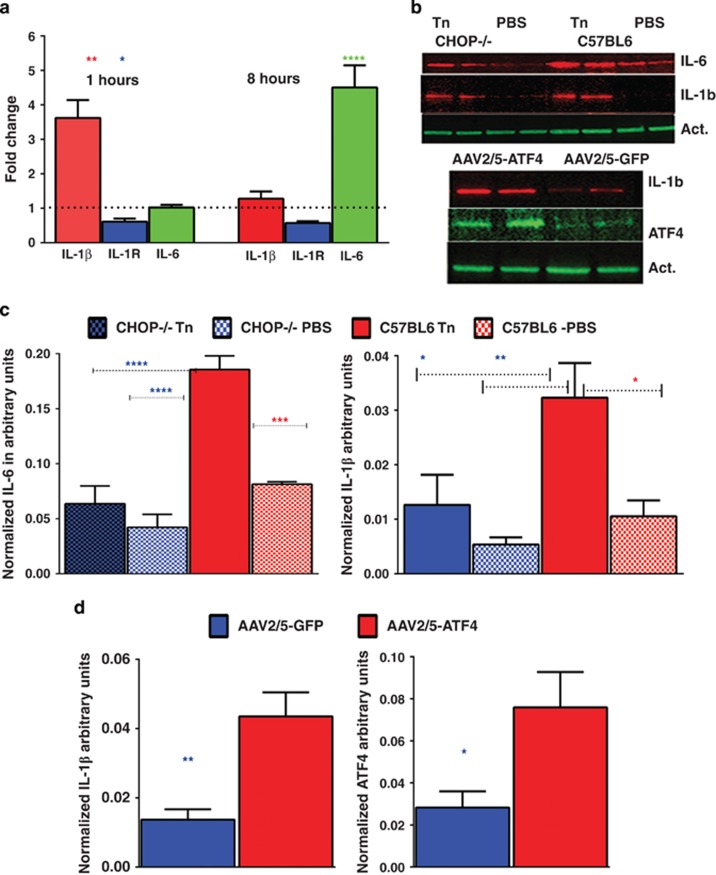
Injection with Tn leads to over production of cytokines in the retinal cells. (**a**) The cone-derived 661W cells treated with Tn (*N*=4) were harvested 1 and 8 h post injection to assess levels of *Il-1β*, *Il-1 R* and *Il-6* by qRT-PCR. Results of the experiments demonstrated that the Tn treatment induces a 3.6-fold upregulation of Il-1*β* mRNA and 33% downregulation of *Il-1*R mRNA 1 h post treatment. (**a**) A 4-fold overexpression of Il-6 mRNA was observed 8 h post treatment with Tn. (**b–d**): Modulation of the UPR markers leads to altered cytokine's production. (**b**) Images of western blots obtained from the CHOP^−/−^ and ATF4 overexpressing retinal extracts. (**c**) CHOP ablation in Tn-injected retinas (*N*=4) leads to a 46% and by 66% reduction in IL-6 and IL-1*β*, respectively, at 3 days post treatment, indicating that CHOP might regulate production of these cytokines. (**d**) The 2.6-fold increase in ATF4 triggers pro-inflammatory IL-1*β* over production. A 3-fold increase in IL-1*β* in AAV2/5 ATF4-injected retinas (*N*=4), suggested that the PERK UPR arm that leads to the ATF4 mRNA increase may be responsible for activation of the IL-1*β* mediated inflammatory signaling. The distribution of data values is shown in S.E.M., *P<0.05, **P<0.01, ***P<0.001 and ****P<0. 0001

Photoreceptors have been reported to express cytokines *Cx3cl1*, *Mcp*-1, *Rantes*, *Il-1β* and *Tnf-α* in response to photo-injury,^16^ a known trigger for UPR activation,^17^ and to release cytokines in response to LPS treatment.^18^ On the basis of this information, we decided to verify whether cone-derived 661W cells induce *Il-1β*, *Il-1R* and *Il*-6 cytokines when treated with Tn and whether modulation of UPR markers would modify cytokine levels in a mouse retina experiencing an ongoing UPR (Figure 2 and Supplementary Table S2). We found that 1 h post treatment 661W cells demonstrated an upregulation of the early-mediated *Il-1β* by 3.6-fold and downregulation of *Il-1R* by 0.67-fold, whereas at 8 h post treatment *Il*-6 mRNA was upregulated by >4-fold. Next, we also analyzed IL-1*β* and IL-6 production in CHOP^−/−^ retinas injected with Tn, as well as in C57BL6 retinas overexpressing ATF4 in their photoreceptors; thus mimicking the activation of the PERK UPR signaling arm. Our results indicated that the ablation of CHOP resulted in a 66% reduction of IL-6 and a 62% of IL-1*β*, in the retinas injected with Tn as compared with Tn-treated C57BL6 controls. These results indicate that CHOP is crucial for IL-6 and IL-1*β* over production.

A 2.6-fold overexpression of ATF4 was achieved in photoreceptors by means of adeno-associated viral (AAV) transduction (serotype 5).^19^ As ATF4 was previously shown to activate IL-6 production,^20^ we concentrated on IL-1*β* and found that it was significantly upregulated by >3-fold in the AAV2/5 ATF4 retinas.

### Retinas of mice with inherited retinal degeneration demonstrate an increase in pro- and anti-inflammatory markers

Previously, we showed that the T17M *RHO* retina expressed hallmarks of the UPR starting from P15, before the onset of any symptoms, and continued to P30 at which point retinal degeneration resulted in a marked loss of photoreceptor cells and vision.^6, 21^ We also demonstrated that the elevation of TNF-*α* in mice expressing T17M *RHO* (rhodopsin class II mutation)^22^ occurred concomitantly with the activation of the TRAF2-pcJun pathway at P30.^23^ These data suggest that the T17M *RHO* retina could experience initiation of inflammatory signaling, perhaps leading to the suppression of pro-survival and elevation of pro-death pathways.

**Figure 3:**
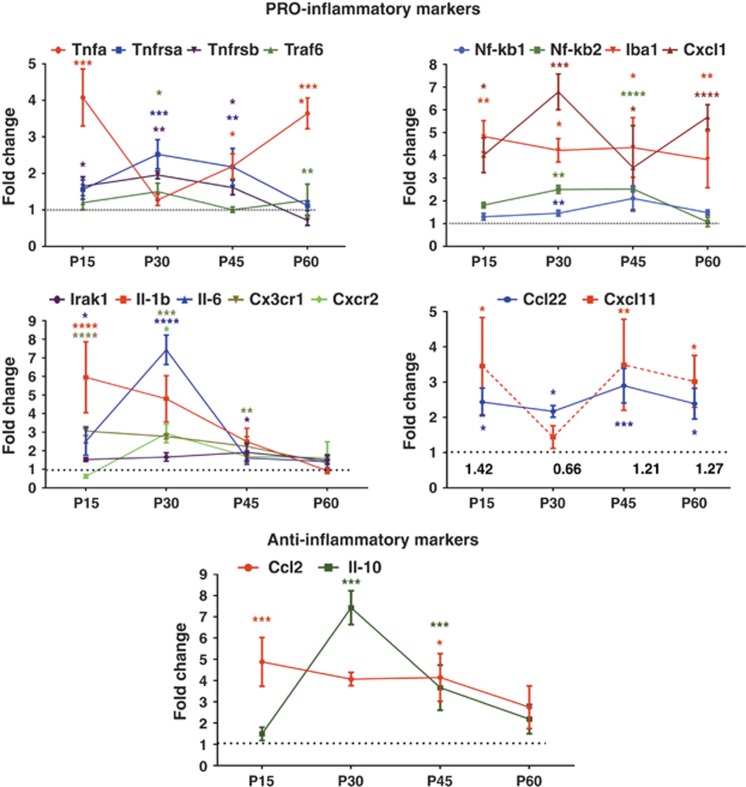
ADRP progression in T17M *RHO* mice was accompanied by activation of the inflammatory response as measured by qRT-PCR (*N*=4). Pro- and anti-inflammatory markers were detected in P15, 30, 45 and 60 ADRP retinas. The distribution of data values is shown in S.E.M., *P<0.05, **P<0.01, ***P<0.001 and ****P<0. 0001

Inflammatory chemokines, interleukins and TNF-*α* can be classified as either pro- or anti-inflammatory biomarkers, but some have more complex, multifunctional roles such as TNF-*α*, TRAF6, IL-1*β* and IL-6. For the sake of simplicity we present our results based on typical pro- and anti-inflammatory classifications of these inflammation biomarkers (Figure 3 and Supplementary Table S3). Western blot analysis and collected qRT-PCR data demonstrate that the expression of both pro- and anti-inflammatory markers changed significantly over the course of ADRP progression in the T17M *RHO* retinas.

We analyzed the P15, P30, P45 and P60 time points and found that mRNAs for the pro-inflammatory markers *Tnfrsb, Il-*1*β*, *Il-*6, *Cx3cr1*, *Cxcl1*, *Tnf-α* and *Iba1* were already significantly upregulated at P15, as compared with wild-type retinas. Their fold change ranged from 1.65- to 6-fold at P15. However, anti-inflammatory *Ccl2/Mcp-1* expression was also significantly increased by 4.9-fold. At P30, in addition to the above mentioned markers, we observed upregulation of the pro-inflammatory *Traf6*, *Nf-kb2*, *Tnfrsα* and *Cxcr2*, which increased by 1.5–2.6-fold. Interestingly, *Il-1β* mRNA was not elevated in the T17M *RHO* retina at any of the time points. The anti-inflammatory *Ccl2/Mcp-1* also did not differ from wild type but *Il-*10 mRNA was elevated by 7-fold in the ADRP retina. At P45, almost all of the examined pro-inflammatory markers were upregulated at the mRNA level. The exceptions were *Traf*6, *Il*-6 and *Cxcr2*. The anti-inflammatory Ccl2 and *Il-*10 mRNAs were elevated by >4- and 3.6-fold, respectively, in the T17M *RHO* retinas. However, by P60 these markers dropped significantly to levels that were no different from controls while pro-inflammatory *Tnf-α*, *Traf*6, *Cxcl*1 and *Iba*1 remained steadily upregulated in the ADRP retina. The observed decrease in anti-inflammatory markers correlated with a significant loss of a- wave ERG amplitude in the T17M *RHO* photoreceptors at P60.^21^

Recently, it has been proposed that cytokines *Cxcl*11 and *Ccl*22 are secreted by M1 and M2 macrophages that are generally considered to be pro- and anti-inflammatory, respectively, and that the ratio of *Ccl*22 to *Cxcl*11 could be used to characterize the polarization of the macrophage population within the advanced AMD retina.^24^ We used these markers to identify these macrophages populations in the retina and found predominantly M1 macrophages during the progression of ADRP. The ratio of *Ccl*22/*Cxc*l11 ranged from 1.2 to 1.4 with the only exception being at P30, when this ratio dropped. This, however, did not result from an increase in M2 macrophages but instead corresponded with a significant drop in *Cxcl*11 mRNA expression.

**Figure 4:**
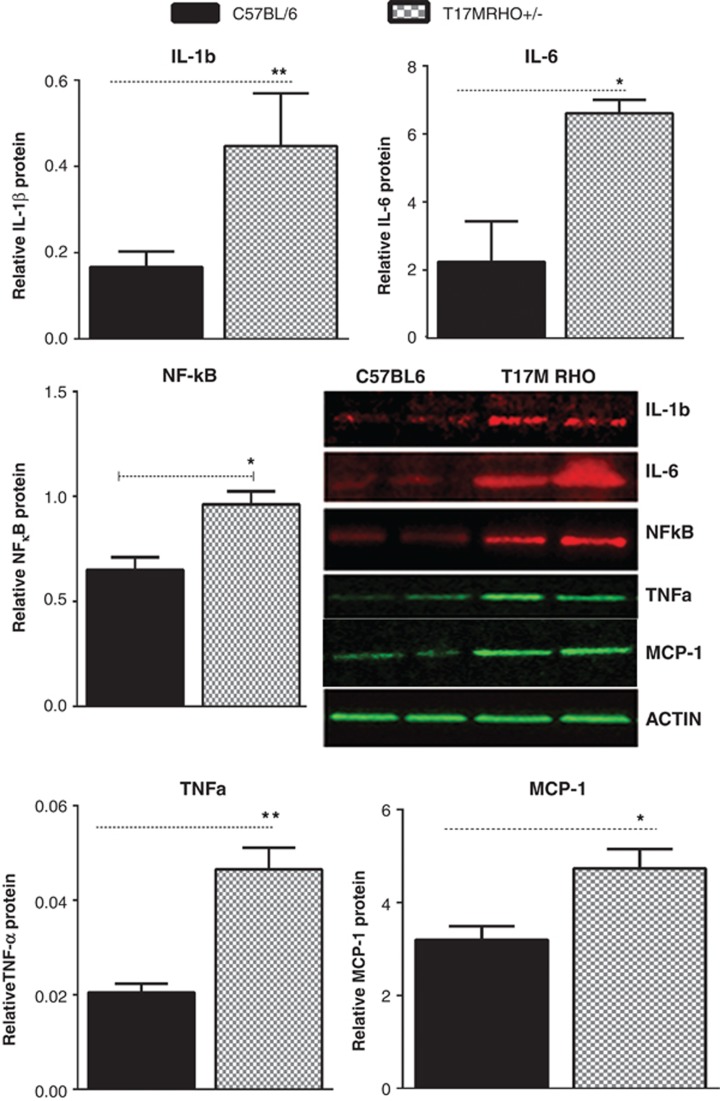
ADRP progression in T17M *RHO* mice is accompanied by activation of the inflammatory response as measured by western blot analysis (*N*=4). IL-1*β*, IL-6, p65 NF-K*β*, MCP-1 and TNF*-α* are significantly upregulated in the P15 ADRP retina (*P*=0.004, *P*=0.002, *P*=0.012, *P*=0.037 and *P*=0.002, respectively). Images of the western blots are shown on the side

Next, we found that the pro-inflammatory proteins IL-1*β*, IL-6, p65 NF-kB, MCP-1 and TNF*-α* were already upregulated in the P15 ADRP retina by 2.7-, 2.9-, 1.5- and 2.3-fold, respectively (Figure 4 and Supplementary Tables S3), whereas the levels of the anti-inflammatory CCL2/MCP-1 increased by 1.5-fold. Thus, western blot analysis confirmed the qRT-PCR data suggesting that both pro- and anti-inflammatory markers were elevated in the P15 ADRP retinas.

Given that both pro- and anti-inflammatory markers were expressed in T17M *RHO* animals, we wanted to determine whether the ADRP retina expressing a class I mutant RHO would have a different inflammatory marker profile. We analyzed the retinas of the knock-in Ter349Glu *RHO* mice, which carry a human rhodopsin gene with a read-through mutation that was generated by adding 51 amino acids to the 1D4 epitope.^25^ The retinas of these mice showed a 50% loss of photoreceptors at 5 weeks (Supplementary Figure S3).

**Figure 5:**
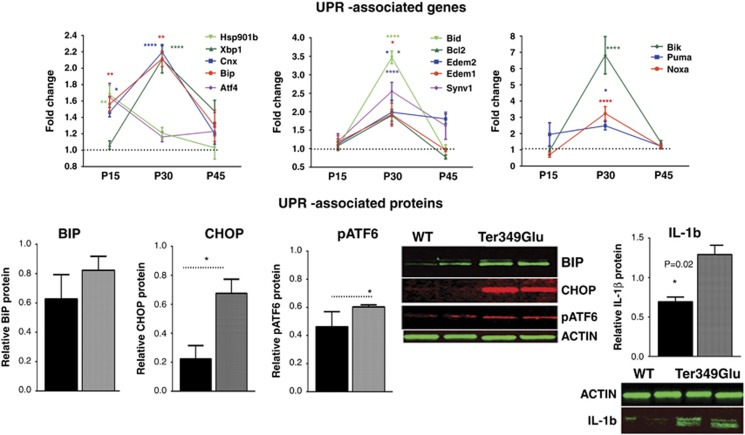
An activated UPR was found in ADRP retinas expressing a class I Ter349Glu *RHO* mutant (*N*=4). The distribution of data values is shown in S.E.M. Upper panel: expression of UPR-associated genes was elevated in Ter349Glu *RHO* retinas during ADRP progression as measured by qRT-PCR. Mainly, overexpression of UPR-associated genes was detected at P30 (*P<0.05, **P<0.01, ***P<0.001 and ****P<0. 0001). Bottom panel: western blot analysis revealed activation of UPR markers CHOP and pATF6 (*P*=0.020 for both) (*N*=4). Images of the western blots are shown on the side. In addition, the inflammatory marker IL-1*β* was found to be increased by 1.9-fold in P30 Ter349Glu retinas (*P*=0.020)

First, we investigated whether or not these mice experienced retinal UPR activation as previously seen in rats carrying another class I rhodopsin mutant^7^ (Figure 5 and Supplementary Table S4). We found that *Bip*, *Cnx*, *Atf4* (PERK signaling) and *Hsp*901b were upregulated by 1.6-fold in the P15 Ter349Glu retinas. At P30, *Bip* mRNA continued to be upregulated by 2-fold. In addition, the *Cnx*, *Xbp*1 (IRE1 signaling), *Edem*1, *Edem*2, *Synovalin*1, *Bcl*2, *Bid*, *Noxa*, *Puma* and *Bik* mRNAs were also upregulated by 2.2-, 2.2-, 1.9-, 1.9-, 2.7-, 1.9-, 3.5-, 3.2-, 2.5- and 6.8-fold, respectively.

Western blot analysis confirmed qRT-PCR results and demonstrated that the hallmarks of UPR activation were upregulated in the P30 Ter349Glu *RHO* retina. Although BIP over production changed slightly in the Ter349Glu *RHO* retina, the CHOP and pATF6 (50) proteins demonstrated a significant increase of over 1.3- and 3-fold, respectively, as compared with controls. In addition, we also found that the UPR activation in the Ter349Glu *RHO* retinas was accompanied by a 1.9-fold increase in the inflammatory marker IL-1*β*.

### Microglia are activated in the ADRP retina expressing either class I and class II mutant rhodopsins

**Figure 6:**
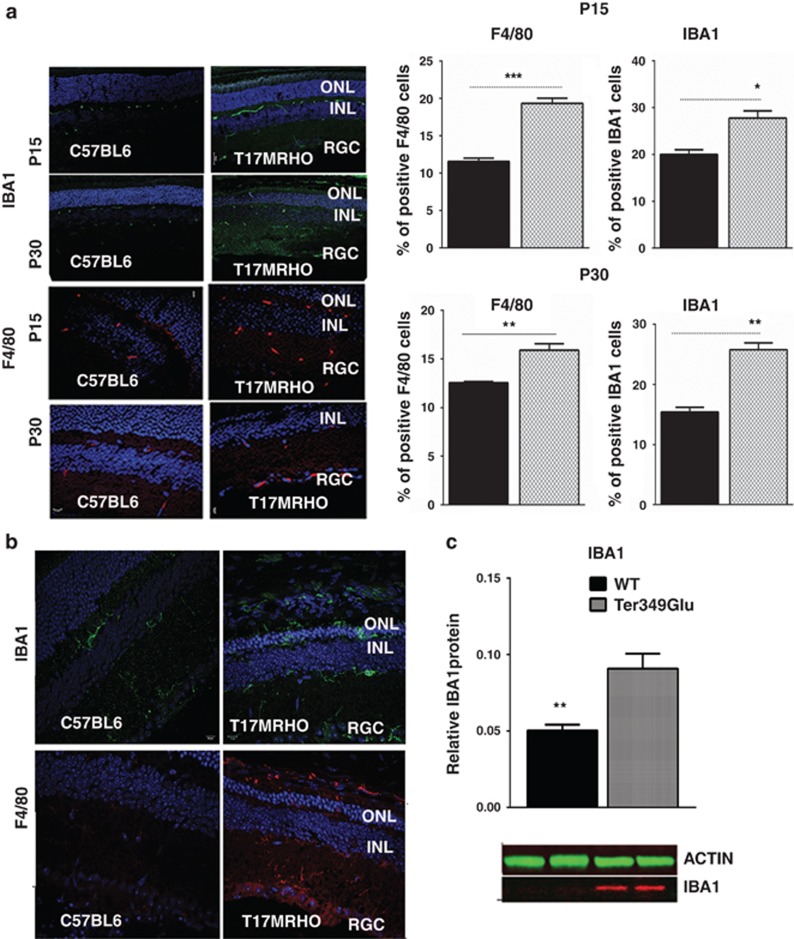
Microglia are activated during ADRP progression. (**a**) The microglial markers F4/80 and IBA1 were used to perform immunohistochemical analysis in P15 and P30 cryostat-sectioned T17M *RHO* retinas (*N*=4). Five images of each individual T17M *RHO* and C57BL6 retina were taken to count positive cells. The number of positive cells was used to plot graphs for statistical analysis. The observed increase in F4/80-positive cells was 67% and 26%, respectively (*P*=0.01 for both time points). In addition, there was an increase in IBA-1 positive cells during ADRP progression from 40–70% compared with controls (*P*=0.013 and *P*=0.002, respectively). The distribution of data values are shown in S.E.M. Representative confocal images of P15 and P30 retinas are shown. (**b**) Activation of the microglial markers IBA1 and F4/80 were found in 10-week-old Ter349Glu retinas. Representative images are shown. (**c**) Almost 2-fold increase in IBA1 protein was found in Ter349Glu retinal protein extracts, *P*=0.014 (*N*=4). Bottom: images of the western blot treated with anti-Iba1 and anti-*β*-actin antibodies

In addition to analyzing the ratio *of Ccl*22/*Cxcl*11, which suggested a trend towards M1 polarization in the ADRP retina, we performed immunohistochemical analysis on the T17M *RHO* retinas and found that the macrophage markers F4/80 and IBA1 were upregulated at P15 and P30, respectively (Figure 6a and Supplementary Table S5). The number of F4/80-positive cells was 1.7- and 2-fold greater than in wild-type retinas at P15 and P30, respectively. The number of IBA1-positive cells was also increased by 1.4- and 1.7-fold. Therefore, the results of immunohistochemical analysis revealed that microglia were activated in the class II mutant rhodopsin expressing retina.

As the Ter349Glu retinas also exhibit UPR activation, we wanted to know whether the microglial activation was also present. We performed western blot and immunohistochemical analysis (Figure 6b) and found that the levels of IBA1 were 1.9-fold higher in the Ter349Glu retina at P30 when compared with wild-type controls. In addition, IBA1 was detected in 10-week-old Ter349Glu retinas by immunohistochemical analysis.

### Injection of recombinant il-1*β* promotes retinal degeneration in the wild-type retina

**Figure 7:**
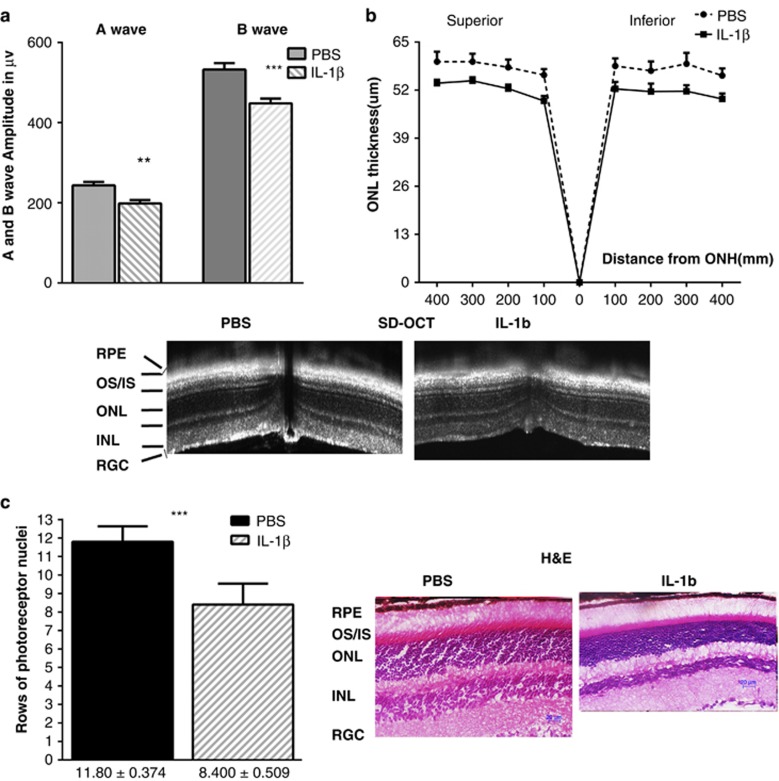
Overexpression of the recombinant IL-1*β* cytokine can induce retinal degeneration in the wild-type retina. (**a**) Intraocular injection of 250 ng of recombinant IL-1*β* led to a statistically significant loss of scotopic ERG amplitudes 30 days after treatment (*N*=6). The a-wave and b-wave amplitudes were significantly reduced (*P*=0.035 and *P*=0.037, respectively). (**b**) SD-OCT analysis confirmed the ERG data indicating a >10% loss of the average ONL thickness in the superior and inferior regions of the IL-1*β*-injected retina (*P*=0.007 and *P*=0.0004, respectively) (*N*=4). Bottom: SD-OCT images of the PBS- and IL-1*β*-injected retinas. (**c**) Histological analysis following H&E staining of cryostat-sectioned IL-1*β*-injected retinas revealed a 29% loss of photoreceptor cells as compared with PBS-injected mice 30 days after treatment (*P*=0.001). Images of H&E stained PBS- and IL-1*β*-injected retinas are shown on the side. Scale bar indicates 20 *μ*m

Given that the T17M *RHO* retinas express pro-inflammatory markers under the conditions of ER stress, like early mediated IL-1*β*, and that the photoreceptors could be the sources of these induced cytokines (Figure 2),^17^ we investigated whether pro-inflammatory cytokines could directly mediate retinal degeneration in the mouse retina. The rationale for this experiment stemmed from the multifunctional nature of many cytokines that are activated during inflammation. In the case of the P15 T17M *RHO* mouse retina, the precise role of IL-1*β* during the course of retinal degeneration is not clear. Figure 7 and Supplementary Table S6 demonstrate results obtained from C57BL6 mice intraocularly injected with recombinant IL-1*β* protein.

We found that at 30 days after injection, a- and b-wave scotopic ERG amplitudes were reduced by 19% and 16%, respectively, suggesting a loss of photoreceptor function. This loss was in agreement with the SD-OCT data demonstrating that the average ONL thickness of the superior and inferior retina, when measured within 400 nm from the optic nerve head (ONH), was reduced by 11% and 12%, respectively. In addition, hematoxylin and eosin (H&E) staining of the IL-1*β*-injected, cryostat sectioned, retinas demonstrated a significant loss (29%) of photoreceptors 30 days after treatment.

## Discussion

A clear understanding of the underlying mechanisms of retinal degeneration has been complicated by the heterogeneity of cases within any particular retinopathy and by the interplay of multiple cellular signaling pathways involved in each disease model.^1^ A recent increase in the number of studies on ER stress in different models of retinal degeneration has significantly advanced our understanding of the mechanisms of retinopathy and highlighted the role of the UPR and individual UPR markers in the pathogenesis of retinal degeneration. However, the main question of whether UPR activation could directly contribute to retinal pathology by promoting retinal degeneration has remained unanswered.

The ER stress occurs in the majority of photoreceptors whose function and vulnerability can be monitored by photoreceptor-originated a-wave scotopic ERG, as well as SD-OCT photoreceptor nuclei layer measurements and H&E staining. Thus, by analyzing a functional loss of photoreceptor cells in the Tn-injected mice, we can evaluate the physiological impact of the UPR on retinal degeneration. In contrast, these relationships would not be possible to track by using the 661W cone-derived cell line.

The dose of 0.001 *μ*g Tn does not cause a persistent activation of UPR and does not induce retinal degeneration as measured by ERG and OCT after 30 days, suggesting a cellular recovery from the transiently activated UPR. It is challenging to produce UPR activation in the retina that both exceeds the physiological norm and does not kill retinal cells.^13, 14, 15^ Monitoring the degree of UPR activation at 3 days post treatment gave us additional confirmation that we were triggering a persistently activated, a mild or possibly a transient UPR. A similar persistently activated UPR was observed in the T17M *RHO* retinas starting at day P15 and continuing through P30.^6^ A strong ER stress response to Tn resulted in the activation of inflammatory cytokines (IL-1*β*, IL-6) and a robust microglial response (MCP-1/CCL2) that was confirmed by an observed increase in the number of IBA-1-positive cells. The UPR-mediated cytokine response could also be signaling by the photoreceptors. Our data and results obtained by others demonstrate that photoreceptors may be the source of the cytokines induced and secreted in the retina.^26^ However, the presence of IL-1R in photoreceptors suggests that IL-1*β* could also be taken up by photoreceptors from the surrounding media. Altogether, overexpression of pro-inflammatory cytokines and UPR-induced proapoptotic CHOP, as well as the elevated microglial response could conceivably ‘kill' wild-type photoreceptors leading to a reduction in the number of detected nuclei in the H&E stained retinal sections.

Among the multitude of potential causes for retinal degeneration by a persistently activated UPR, the activation of inflammatory signaling is especially intriguing. The modulation of CHOP and ATF4 in the mouse retinas demonstrates a regulatory role for these UPR markers in IL-6 and IL-1*β* production and confirms results from previous studies on the activation of IL-1*β* and IL-6 via the ER stress CHOP pathway, as well as the ATF4-mediated increase in IL-6.^20, 27, 28^ The spectrum of ocular disease associated with dysfunction of the immune system has continued to expand over the past decade.^29, 30, 31, 32, 33, 34, 35^ However, to date there has been no definitive evidence of a significant causal role for an inflammation in retinal degeneration. Thus, based on the finding of Tn-induced IL-1*β* expression, we examined the role of pro-inflammatory IL-1*β* in the wild-type retina to determine whether the overexpression of this cytokine could promote progressive retinal degeneration.

Previously examined electrophysiological effect of recombinant IL-1*β* on the rabbit retina demonstrated that IL-1*β* produced a pathophysiological impact.^36^ However, the delay in the visual evoked potential seen in injected eyes was found to be reversible 24 and 48 h after injection. In our experiment, the pathophysiological effect of IL-1*β* was evaluated 30 days post injection and we observed a statistically significant reduction of the photoreceptor-originated a-wave scotopic ERG amplitude. Moreover, the photoreceptor functional loss was in agreement with the reduction in the number of photoreceptor cells suggesting a pathophysiological role for the pro-inflammatory IL-1*β* in the retina. Even though the loss of photoreceptor function in the IL-1*β*-injected retinas was not overly profound (19%), it is necessary to keep in mind that in a real diseased condition, IL-1*β* would not be the only pro-inflammatory cytokine overexpressed in the retina. The mechanism by which IL-1*β* exerts cytotoxicity still needs to be studied. We cannot rule out the possibility that in real-world disease conditions such as ADRP, IL-1*β* is released in the degenerating retina by infiltrating microglia^37^ in response to other pro-inflammatory cytokines released by photoreceptors in a UPR-triggered manner. However, in either the case of IL-1*β* release or the uptake by photoreceptors, it is capable of promoting photoreceptor cell death, by a mechanism that can be associated with multiple signaling pathways including induced neovascularization.^38^ Therefore, we believe that one possible way to trigger retinal degeneration in mice with an activated UPR is to induce overexpression of pro-inflammatory cytokines in the photoreceptors.

Persistent UPR activation in T17M *RHO* retinas^6^ correlates with the rate of retinal degeneration^21^ and the production of pro-inflammatory markers. Expression of the class II mutant T17M *RHO* in the mouse retina resulted in the consistent upregulation of pro-inflammatory markers such as IL-1*β* and IL-6 from P15, suggesting a strong pro-inflammatory response at the onset of ADRP. The anti-inflammatory response was also activated at this time. Likewise, the CCL2/MCP-1 protein was significantly upregulated at P15 but the mRNA was significantly downregulated at P60.

P30 seemed to be a critical time point for ADRP progression and was characterized by marked photoreceptor functional loss and cell death.^21^ The level of anti-inflammatory *Il-10* mRNA increased at this point suggesting a polarization towards M2 macrophages, which are known to be capable of promoting CNS repair.^39^ This potential enhancement in the M2 population of the P30 ADRP retina was supported by the observed 1.5-fold increase in the ratio of *Ccl*22/*Cxcl*11 mRNAs.^24^ However, at P15, P45 and P60, the M1 phenotype appeared to be predominant and this may in fact have contributed to ADRP progression.

Unsurprisingly, mice expressing the class I mutant Ter349Glu *RHO* also demonstrated the UPR induction at P30, when animals demonstrated a 50% loss of photoreceptor cells. We have previously demonstrated that the retinas expressing the S334ter *Rho* experienced UPR activation.^7^ Together, these studies imply that the ER homeostasis in photoreceptors expressing class I RHO mutants may be generally compromised. Although a Ter349Glu *RHO* mutant is not misfolded in these mice, a variety of stimuli including disturbances in redox regulation, calcium regulation, glucose deprivation and viral infection can compromise ER homeostasis. Consequently, by analogy with the T17M *RHO* retinas, we analyzed the inflammatory markers and discovered that class I RHO mutants induced an increase in IL-1*β*, thus suggesting that UPR and inflammatory activation occurred concomitantly in both the ADRP models. As with T17M *RHO* photoreceptors, the Ter349Glu *RHO* photoreceptors could also be the source of the secreted cytokines, resulting in the observed F4/80 and IBA1 microglia increase within the retinas.

Our results indicate that a persistently active UPR signaling in ADRP photoreceptors can promote retinal degeneration. In addition to the UPR-stimulated TNF*-α* induction, which in T17M *RHO* retinas is accompanied by activation of JNK signaling,^23^ UPR-induced retinal degeneration might also be provoked by induction of pro-inflammatory cytokines like IL-1*β*. Therefore, for the first time, our data link UPR activation with inflammation and retinal degeneration seen in the diseased retinas. Our findings also highlight a potential mechanism responsible for ocular disorders that may not be directly associated with protein misfolding as a primary cause for UPR activation.

## Materials and Methods

### Animals and treatments

C57BL/6, T17M *RHO*^+/−^, CHOP^−/−^, ERAI^+/−^, C57BL/6 and albino Ter349Glu *RHO* knock-in mice were obtained from our animal facility housed in an air-conditioned room (23±1 °C) under 12 h dark/12 h light cycles. Ter349Glu *RHO* knock-in mice were created as previously described.^25^ The animal protocol was approved by the University of Alabama at Birmingham Institutional Animal Care and Use Committee and was conducted following the animal guidelines according to the ARVO statement for the Use of Animals in Ophthalmic and Vision Research. The genotype was confirmed using PCR analysis as previously described.^6, 21^ Retinas were collected from individual groups and wild-type animals at postnatal days 15, 30, 45 and 60 for our study.

### Subretinal injection with AAV

Subretinal injections were performed in pups at postnatal day 15 with 1 *μ*l of either AA2/5 virus expressing the mouse ATF4 complementary DNA (cDNA) or GFP (10^13^ genome particle per ml for both viruses) under the control of the CMV enhancer–chicken *β*-actin promoter. Viral vector was injected into the right eye and PBS in the left eye.^6^ Animals were monitored for 2 weeks. Retinal protein analysis of injected retinas were performed to evaluate the results of ATF4 overexpression.

### Preparation of IL-1*β* human recombinant protein and Tn solution

IL-1*β* and Tn for the treatment groups, or PBS for control groups, were injected intraocularly in C57BL/6 mice. IL-1*β* human recombinant protein (Cat # GWB-267F10) was injected at a concentration of 250 ng per eye (125 ng/*μ*l, 2 *μ*l) (Planck *et al.*^40^). Tn (Cat# T7765 Sigma-Aldrich, St Louis, MO, USA) from *Streptomyces* was dissolved in water at a concentration of 1 mg/ml, 2 *μ*l (0.01 *μ*g/*μ*l concentrations)^15^ was injected for each mouse eye. PBS was used as a control and was injected into the other eye of each animal.

### Quantitative real-time RT-PCR

The retina was placed in TRIzol reagent (Invitrogen, Carlsbad, CA, USA) to extract the total RNA. cDNA was made by adding 1*μ*g of the total RNA to the High Capacity RNA-to-cDNA Master Mix (Applied Biosystems, Foster City, CA, USA), and reverse transcribing according to the manufacturer's instructions. PCR was performed at 50 °C for 2 min and 95 °C for 10 min, followed by 40 cycles at 95 °C for 15 s and 60 °C for 1 min. The mRNA levels of these genes were normalized to that of *Gapdh*. We used a custom Taqman array plate with 22 genes, including *Gapdh* as an endogenous control (Applied Biosystems, Carlsbad, CA, USA). qRT-PCR was performed using 50 ng of cDNA mixed with TaqMan universal PCR master mix (Applied Biosystems) in the StepOnePlus Real-Time PCR system (Applied Biosystems, *N*=4 for each time point). The fold changes were calculated by dividing the mean of the relative quantities (RQs) for the T17M *RHO* mice by the mean RQ of the wild-type mice at each time point. The results were analyzed by two-way ANOVA using GraphPad Prism (GraphPad Software, Inc., La Jolla, CA, USA).

### Cell culture

The 661W photoreceptor cell line was generously provided by Dr Muayyad Al-Ubaidi (Department of Cell Biology, University of Oklahoma Health Sciences Center, Oklahoma City, OK, USA). These cells were cultured in Dubelcco's modified Eagle's medium (Invitrogen) supplemented with 10% heat-inactivated fetal bovine serum and 1% penicillin/streptomycin (Invitrogen), at 37 °C in a humidified atmosphere with 5% CO_2_. Cells (10^6^) were seeded in tissue culture 100 mm Petri dish and were allowed to attach for 15 h. The cells were then washed with PBS twice (pH 7.4) followed by the addition of 10 ml serum-free medium. The control group contained only media, whereas the experimental group was treated with Tn (10 *μ*g/ml) for 1 or 8 h. After incubation at different time points cells were harvested, washed with PBS and collected for RNA isolation. cDNA preparation was described previously. qRT-PCR was performed using 66 ng of cDNA, TaqMan Universal PCR master (Applied Biosystems) and the StepOnePlus Real Time PCR system (*N*=4). We detected *Il-1R, Il-1β and Il-6* genes including *Gapdh* as a control. The fold change was calculated by dividing the mean of the RQs for the control by the mean RQ of the Tn treatment at each time point. The result were analyzed by one-way ANOVA using GraphPad Prism software.

### Western blots (WB)

Retinas were rinsed in lysis buffer (NP-40, 50 mM Tris, 150 mM NaCl, 1% Triton X-100) and halt protease and phosphatase inhibitor cocktail (Prod# 1861281, ThermoScientific, Rockford, IL, USA). Protein concentration was determined using the Bradford method (Bradford, MM 1976). Total protein (40 *μ*g per well) was electrophoresed on a 4–12% SDS-polyacrylamide gradient gel (Bio-Rad, Hercules, CA, USA) and blotted onto a PVDF membrane (Cat # 170-4157). The blot was incubated with primary antibody overnight at 4 °C, washed and incubated with the secondary antibody for 1 h at room temperature. The bands were visualized using either the enhanced chemiluminescence detection system (Western Sure Ultra Chemiluminescent Substrate parts; Cat #926-85000, LI-COR, Inc., Lincoln, NE, USA) or infrared secondary antibodies and an Odyssey infrared imager (LI-COR Model: 2800 S/N OFC-0172).

Antibodies used were as follows: anti-TNF*-α* mouse monoclonal (1 : 1000) (Cat#ab1793, Abcam, Cambridge, MA, USA); anti-IL-1*β* rabbit monoclonal (1 : 1000) (Cat# 12507, Cell signaling); anti-NFKB p65 rabbit polyclonal (1 : 1000) (Cat# 06-418, Millipore, Billerica, MA, USA); anti-MCP-1 goat polyclonal (1 : 1000) (Cat# Sc1784 Santacruz); IL-6 rabbit polyclonal (1 : 1000) Cat# Ab6672, Abcam); anti-ATF6 rat monoclonal (1 : 1000) (Cat# ab6160, Abcam); anti-CHOP mouse monoclonal (1 : 300) (Cat# Sc7351, Santa-Cruz Biotechnology, Santa-Cruz, CA, USA); anti-Phosphorylated eIF2*α* rabbit monoclonal (1 : 1000) (Cat# 3398 Cell Signaling, Danvers, MA, USA); anti-TNF*-α* (1 : 1000) and anti-Bip goat polyclonal (1 : 300) (Cat# Sc1050, Santa-Cruz Biotechnology) and anti-*β*-actin mouse monoclonal (1 : 1000) (Cat# A1978, Sigma-Aldrich), anti-tubulin rat monoclonal (1 : 1000) (Cat# ab6160, Abcam). Donkey anti-goat IRDye (1 : 10 000) (Cat# 926-32214, LI-COR); goat anti-mouse IRDye (1 : 10 000) (Cat# 926-32210 LI-COR, Inc.); goat anti-rat IRDye (1 : 10 000) (Cat# 926-32229, LI-COR, Inc.); HRP-conjugated goat anti-rabbit IgG (1 : 20,000) (Cat #31460, ThermoScientific, Waltham, MA, USA). The results were analyzed by two-way ANOVA using GraphPad Prism (GraphPad Software, Inc.).

### Histological analysis

Mice were killed using a CO_2_ chamber. The eye balls were enucleated, afixed in 4% freshly made paraformaldehyde (Cat# S898-09 J.T.Baker, Phillipsburg, NJ, USA) and kept at 4 °C for 8 h. Then, eyecups were transferred to fresh PBS to remove formaldehyde and immersed in a 30% sucrose solution for cryoprotection. Eyecups were then embedded in cryostat compound (Tissue TEK OCT, Sakura Finetek USA, Inc., Torrance, CA, USA) and frozen at −80 °C. Twelve-micron sections were obtained using a cryostat. To count the nuclei of photoreceptors, we stained cryostat-sectioned retinas with H&E using an H&E stain Kit (Cat#3490). Other slides were used for immunohistochemistry. Digital images of the right and left retinas of individual mice were taken and the outer segment length was analyzed in the central superior and inferior retina, located equidistant from the ONH. Images were analyzed by a blinded investigator. All sections were examined on a microscope equipped with a digital camera (Carl Zeiss Axioplan2 Imaging microscope B000707, Carl Zeiss, Gottingen, Germany).

### Immunohistochemical analysis

Twelve-micron sections were obtained and fixed on polylysine-treated glass slides. Slides were warmed for 30 min at 37 °C and washed in 0.1 M PBS for 10 min three times. Slides were kept in blocking buffer with 10% normal goat serum and 0.3% Triton solution for 1 h at room temperature and washed with PBS three times. The sections were incubated with primary antibodies at 4 °C overnight. The slides were then washed three times with PBS and incubated with secondary antibody for 1 h at room temperature. After washing, mounting medium was added to slides containing DAPI, and was allowed to dry for 1 h. Images were taken using a confocal microscope.

The following primary antibodies were used for immunohistochemical analysis: Anti-F4/80 rat monoclonal (1 : 50), Anti-IBA1 rabbit (0.5 *μ*g/ml) (Cat#019-19741 Wako Chemicals); and secondary anti-mouse antibody (1 : 10 000); the anti-rat antibody (1 : 10 000). Images of retinas were obtained using confocal microscopy.

### Electroretinogram

Mice were dark adapted for at least 12 h, and anesthetized by intraperitoneal injection of 50 mg xylazine and ketamine/kg body weight. The mouse corneas were anesthetized locally with 0.5% proparacaine hydrochloride (Bausch & Lomb, Rochester, NY, USA), and the pupils were dilated with 2.5% phenylephrine hydrochloride (Bausch & Lomb). The ground and reference electrodes were inserted subcutaneously in the hind limb and centered along the nasal ridge, respectively. Gold loop electrodes were placed on each eye. The amplitude of the a-wave was measured from the baseline to the trough of the a-wave, and the amplitude of the b-wave was measured from the trough of the a-wave to the peak of the b-wave. The scotopic ERGs were performed on IL-1*β-*treated group and Tn-treated mice after day 10 and day 30 using LKC Technologies Bigshot Ganzfeld Stimulator, Gaithersburg, MD, USA, as previously described^23^ and were registered with 10 *μ*s flashes of white light at −20, −10, 0, 5, 10 and 15 db. PBS injection was used as control for both the treatment groups (IL-1*β* and Tn).

### Spectral domain optical coherent tomography

The SD-OCT measurements were performed at P30, P60 and P90 using the Spectral Domain Ophthalmic Imaging System (SDOIS) (Bioptigen, Morrisville, NC, USA) on anesthetized mice. Horizontal volume scans through the area dorso-temporal from the optic nerve (superior retina) and the area ventro-temporal from the optic nerve (inferior retina) were used to evaluate the thickness of the ONL. For measuring the thickness of the ONL, six calibrated calipers were placed in the superior and inferior hemispheres of the retinas within 100, 200, 300 and 400 *μ*m of the ONH. The thickness of the ONL was determined by averaging ten measurements.

### Statistical analysis

A one- and two-way ANOVA was used to assess statistical significance for the gene expression assays, as well as the ERG and OCT analyses. The paired *t*-test was used to calculate differences in protein levels, number of F4/80- and Iba1-positive cells and the number of photoreceptor rows in the retina. Data are reported as mean±S.E.M. For all experiments, a *P*-value <0.05 was considered significant (*P<0.05, **P<0.01, ***P<0.001 and ****P<0.0001).

## Abbreviations

ERG: electroretinography
SD-OCT: spectral domain optical coherence tomography
UPR: unfolded protein response
IL-1*β*: Interleukin-1*β*
TNF-*α*: tumor necrosis factor-*α*
MCP-1: monocyte chemoattractant protein-1
NF-kB: nuclear factor kappa B
ER: endoplasmic reticulum
ADRP: autosomal dominant retinitis pigmentosa
RHO: rhodopsin
ERAI: ER stress activated indicator
Tn: tunicamycin
ONL: outer nuclear layer
H&E: hematoxylin and eosin
ONH: optic nerve head
