# Supplementary figures and images for: An activated unfolded protein response promotes retinal degeneration and triggers an inflammatory response in the mouse retina
Source: Cell Death Dis. 2014 Dec 18;5(12):e1578–. doi: 10.1038/cddis.2014.539 (PMC4454166; doi:10.1038/cddis.2014.539)

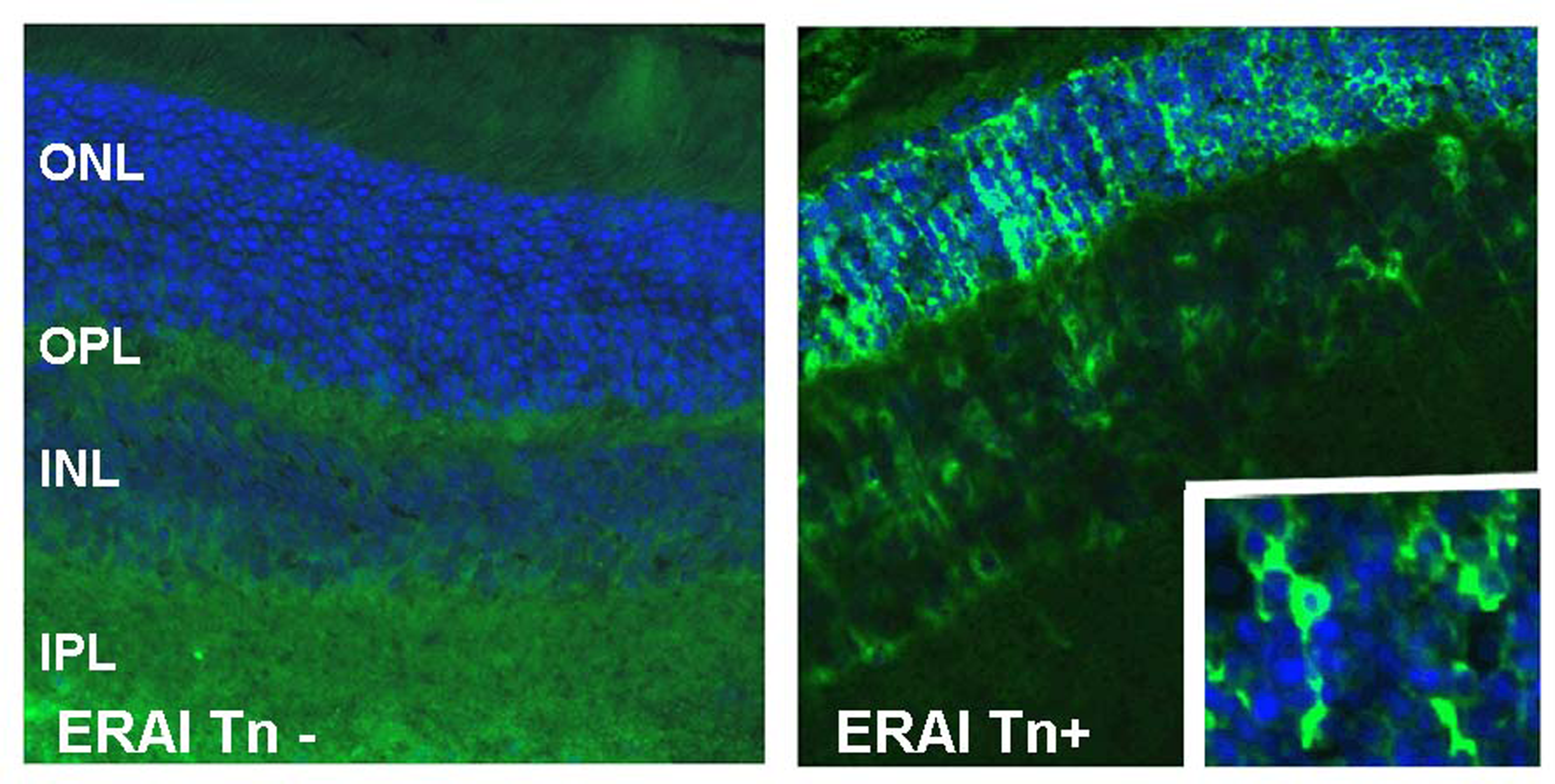

Supplement: Supplementary Figure 1 [file cddis2014539x1.tif]

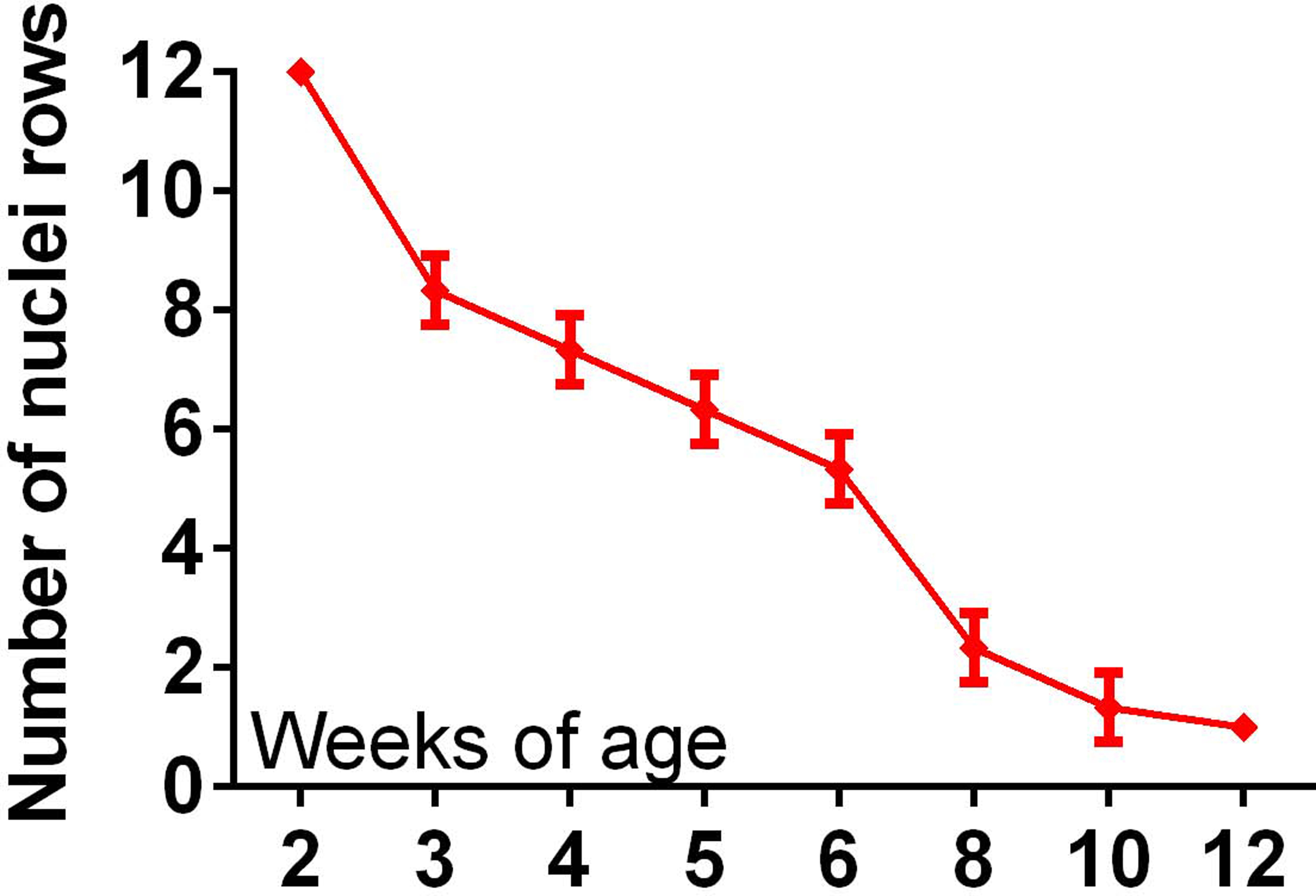

Supplement: Supplementary Figure 3 [file cddis2014539x3.tif]
