# Supplementary material for: An activated unfolded protein response promotes retinal degeneration and triggers an inflammatory response in the mouse retina
Source: Cell Death Dis. 2014 Dec 18;5(12):e1578–. doi: 10.1038/cddis.2014.539 (PMC4454166; doi:10.1038/cddis.2014.539)
Supplement: Supplementary Figure Legends [file cddis2014539x4.doc]

Figure S1. Injection of the ERAI retinas with Tn leads to activation of the UPR, XBp1splicing and production of GFP in cells with ongoing UPR. Majority of the ERAI photoreceptors demonstrated the activated UPR and the GFP expression observed by fluorescent microscopy 24 hour post injection. In addition, other retinal cells were GFP-positive. The GFP expression was observed in OPL (Outer Plexiform layer), INL (Inner Nuclear Layer) and IPL (Inner Plexiform Layer) indicating that horizontal, bipolar, amacrine and Muller glia cells could be also experienced the activated UPR.

Figure S2**.** Injection of wild type retinas with Tn leads to activation of the microglial marker. A >2-fold increase in IBA1 positive cells was found at day 3 post treatment.

Figure S3. Photoreceptor cell loss in Ter349Glu^+/+^ retinas.
