# Supplementary material for: An activated unfolded protein response promotes retinal degeneration and triggers an inflammatory response in the mouse retina
Source: Cell Death Dis. 2014 Dec 18;5(12):e1578–. doi: 10.1038/cddis.2014.539 (PMC4454166; doi:10.1038/cddis.2014.539)
Supplement: Supplementary Table 1 [file cddis2014539x5.pdf]

**Table S1. Tn-induced Unfolded Protein response promotes retinal degeneration in the wild type retina.**

**Western Blot: UPR markers and Inflammatory markers**

| Protein | PBS | Tunicamycin (Tn) | Ratio of Tn/PBS |
|---|---|---|---|
| ATF6 | 0.200 ± 0.055 | 0.411 ± 0.0368 | 2.05 |
| CHOP | 6.919 ± 0.378 | 9.344 ± 0.618 | 1.33 |
| BIP | 0.0488 ± 0.0170 | 0.047 ± 0.002 | 0.96 |
| IL-6 | 1.589 ± 0.0954 | 3.220 ± 0.481 | 2.02 |
| MCP-1 | 0.0658 ± 0.0153 | 0.132 ± 0.013 | 2.00 |
| TNF-ALPHA | 0.051 ± 0.0007 | 0.081 ± 0.004 | 1.56 |
| PeIF2alpha | 0.045 ± 0.002 | 0.173 ± 0.022 | 3.83 |
| IL-1b | 0.946 ± 0.002 | 1.919 ± 0.057 | 2.02 |

**ERG results: 10 and 30 days after the treatment**

| ERG | C57BL/6-d10 | PBS-d10 | TUN- d10 | C57BL/6-d30 | PBS-d30 | TUN- d30 |
|---|---|---|---|---|---|---|
| A-wave | 304.6±20.59 | 281.5±12.79 | 96.83±2.8 | 299.3±43.48 | 303.3±23.77 | 119.3±20.59 |
| B-wave | 659.4±54.22 | 660.4±63.45 | 313.5±14.63 | 620.7±63.48 | 623.1±50.86 | 360.7±45.79 |

**SD-OCT data: 30 days after the treatment**

| OCT ON DAY 30 | PBS | Tunicamycin (Tn) | Ratio of Tn/PBS |
|---|---|---|---|
| Superior | 59.26 ± 0. 323 | 38.85 ± 0.568 | 0.65 |
| Inferior | 60.14 ±0.25 | 38.16±0.47 | 0.63 |
