# Supplementary material for: An activated unfolded protein response promotes retinal degeneration and triggers an inflammatory response in the mouse retina
Source: Cell Death Dis. 2014 Dec 18;5(12):e1578–. doi: 10.1038/cddis.2014.539 (PMC4454166; doi:10.1038/cddis.2014.539)
Supplement: Supplementary Table 2 [file cddis2014539x6.pdf]

Table 2. Treatment of 661W cells with Tn.

GENE	1HR		8 HR	
	CTRL	Tn	CTRL	Tn
IL1R	1.078 ± 0.040	0.721 ± 0.091	0.801 ± 0.14	0.368 ± 0.140
IL1b	0.217 ± 0.045	0.786 ± 0.11	0.203 ± 0.058	0.514 ± 0.083
IL6	0.849 ± 0.081	0.866 ± 0.067	0.947 ± 0.11	3.934 ± 0.6364

Intraocular injection of $CHOP^{-/-}$ mice with Tn (0.010 µg/eye)

Protein	$CHOP^{-/-}$ +TN	$CHOP^{-/-}$ +PBS	C57BL6+Tn	C57BL6+PBS	$CHOP^{-/-}$+Tn vs C57BL6+Tn	$CHOP^{-/-}$+Tn vs $CHOP^{-/-}$ +PBS	$CHOP^{-/-}$+PBS vs. C57BL6+PBS
IL6	0.063± 0.016	0.042± 0.011	0.185 ± 0.012	0.081 ± 0.002	0.34	1.5	0.52 (NS)
IL1b	0.012± 0.005	0.005± 0.001	0.032 ± 0.006	0.010 ± 0.003	0.375	2.4	0.5

Subretinal injection of AAV2/5 ATF4 in C57BL6 retinas.

Protein	AAV2/5-GFP	AAV2/5-ATF4
IL1B	0.013 ± 0.003	0.043 ± 0.006
ATF4	0.028 ± 0.007	0.075 ± 0.016
