# Supplementary material for: An activated unfolded protein response promotes retinal degeneration and triggers an inflammatory response in the mouse retina
Source: Cell Death Dis. 2014 Dec 18;5(12):e1578–. doi: 10.1038/cddis.2014.539 (PMC4454166; doi:10.1038/cddis.2014.539)
Supplement: Supplementary Table 3 [file cddis2014539x7.pdf]

Table S3. T17M RHO retinas experience the activation of inflammatory signaling.

Pro-inflammatory markers

Genes	C57BL/6-P15	C57BL/6- P30	C57BL/6-P45	C57BL/6-P60	T17MRHO+/-: P15	T17MRHO+/-: P30	T17MRHO+/-: P45	T17MRHO+/-: P60
**TNF-ALPHA**	**1.030± 0.0719**	**0.5343± 0.242**	**1.671± 0.385**	**1.911± 0.319**	**4.201± 0.804**	**0.6827± 0.081**	**3.668± 0.571**	**6.965± 0.804**
**NFKB2**	**0.878± 0.063**	**0.778± 0.085**	**1.435± 0.148**	**1.088± 0.063**	**1.592± 0.104**	**1.949± 0.133**	**3.053± 0.580**	**1.169± 0.221**
**NFKB1**	**1.209± 0.093**	**1.088± 0.048**	**1.059± 0.020**	**1.120± 0.062**	**1.577± 0.175**	**1.589± 0.134**	**2.233± 0.589**	**1.665± 0.130**
**CX3CR1**	**0.885± 0.055**	**0.8429± 0.066**	**1.031± 0.076**	**1.256± 0.105**	**2.704± 0.211**	**2.336± 0.105**	**2.305± 0.539**	**1.683± 0.323**
**TNFRS-b**	**0.853± 0.140**	**0.8287± 0.119**	**0.9898± 0.072**	**1.140± 0.062**	**1.407± 0.218**	**1.623± 0.085**	**1.600± 0.195**	**0.8145± 0.157**
**TNFRS-a**	**1.099± 0.068**	**0.9399± 0.057**	**1.015± 0.026**	**1.140± 0.093**	**1.716± 0.291**	**2.372± 0.375**	**2.210± 0.513**	**1.271± 0.149**
**CXCL1**	**0.855± 0.056**	**0.7983± 0.084**	**1.536± 0.563**	**1.181± 0.064**	**3.429± 0.653**	**5.424± 0.628**	**5.325± 2.826**	**6.712± 0.636**
**TRAF6**	**1.118± 0.095**	**1.032± 0.028**	**1.078± 0.026**	**0.9823± 0.140**	**1.340± 0.214**	**1.553± 0.236**	**1.086± 0.079**	**1.656± 0.185**
**Iba1**	**0.972± 0.034**	**0.7874± 0.086**	**0.8482± 0.089**	**1.150± 0.062**	**4.700± 0.669**	**3.326± 0.402**	**3.686± 1.110**	**4.395± 1.426**
**IRAK1**	**0.9870± 0.076**	**0.9094± 0.071**	**0.9741± 0.066**	**1.166± 0.094**	**1.508± 0.107**	**1.513± 0.207**	**1.863± 0.476**	**1.750± 0.268**
**IL-BETA1**	**1.518± 0.513**	**0.5730± 0.153**	**0.9771± 0.132**	**1.609± 0.412**	**9.041± 2.896**	**2.748± 0.714**	**2.440± 0.696**	**1.483± 0.248**
**IL-6**	**1.628± 0.319**	**0.932± 0.043**	**1.456± 0.260**	**0.6158± 0.384**	**4.079± 1.200**	**6.932± 0.741**	**2.350± 0.512**	**0.871± 0.233**
**CXCR2**	**1.733± 0.4531**	**0.668± 0.223**	**0.483± 0.268**	**1.004± 0.173**	**1.079± 0.173**	**1.958± 0.333**	**0.8204± 0.156**	**1.641± 0.854**
**Cxcl11**	**1.078± 0.124**	**0.871± 0.299**	**1.031± 0.228**	**1.107± 0.488**	**3.724 ± 1.485**	**1.051± 0.286**	**3.598± 1.329**	**3.341± 0.8123**
**Ccl22**	**0.9157± 0.07966**	**0.9015± 0.06993**	**0.9162± 0.1129**	**0.8111± 0.08114**	**1.968± 0.3652**	**1.956± 0.1491**	**2.655± 0.4488**	**1.937± 0.3523**

Anti-inflammatory markers

Genes	C57BL/6- P15	C57BL/6- P30	C57BL/6- P45	C57BL/6- P60	T17MRHO+/-: P15	T17MRHO+/-: P30	T17MRHO+/-: P45	T17MRHO+/-: P60
**CCL2**	**1.864± 0.344**	**0.796± 0.108**	**1.456± 0.251**	**1.084± 0.320**	**9.096± 2.137**	**3.240± 0.249**	**6.036± 1.631**	**2.979± 1.083**
**IL-10**	**1.124± 0.082**	**0.9329± 0.043**	**1.736± 0.250**	**1.026± 0.159**	**1.678± 0.347**	**6.932± 0.741**	**6.364± 1.842**	**2.244± 0.7063**

T17MRHO western blot

Protein	C57BL/6- P15	C57BL/6- P30	T17MRHO+/-: P15	T17MRHO+/-: P30
**Il1-beta**	**0.1668 ± 0.01809**		**0.4470 ± 0.06101**	
**IL-6**	**2.240 ± 1.194**	**6.589 ± 1.363**	**6.608 ± 0.228**	**2.130 ± 0.852**
**MCP-1**	**3.201 ± 0.291**	**3.895 ± 0.5815**	**4.737 ± 0.414**	**2.955 ± 0.492**
**P65NF-KB**	**0.649 ± 0.060**	**0.768 ± 0.047**	**0.9610 ± 0.063**	**0.633 ± 0.104**
**TNF-ALPHA**	**0.020 ± 0.0013**	**0.019 ± 0.0020**	**0.04651 ± 0.004**	**0.017 ± 0.0022**
