# Supplementary material for: An activated unfolded protein response promotes retinal degeneration and triggers an inflammatory response in the mouse retina
Source: Cell Death Dis. 2014 Dec 18;5(12):e1578–. doi: 10.1038/cddis.2014.539 (PMC4454166; doi:10.1038/cddis.2014.539)
Supplement: Supplementary Table 4 [file cddis2014539x8.pdf]

Table S4. Unfolded Protein Response is activated in Ter349Glu RHO retina.

qRT-PCR data

GENES	Wild type/6- P15	Wild type/6- P30	Wild type/6- P45	TER349GLY/-: P15	TER349GLY/-: P30	TER349GLY+/-: P45
PUMA	1.055±0.072	1.525±0.327	0.884±0.080	2.064±0.752	3.793±0.393	1.180±0.104
BCL2	0.984±0.116	0.633±0.171	0.850±0.077	1.066±0.118	1.205±0.102	0.702±0.049
BIK	0.746±0.150	0.479±0.219	1.092±0.343	0.712±0.189	3.271±0.5498	1.569±0.144
BID	0.889±0.137	2.073±0.540	0.842± 0.163	0.926±0.086	7.195±0.348	0.782±0.148
ATF4	0.805±0.093	0.7549±0.116	0.9088±0.0937	1.326±0.137	0.8782±0.045	1.119±0.045
GRP94	0.887±0.150	0.7976±0.0801	0.974±0.0267	1.488±0.100	0.9641±0.054	1.003±0.134
XBP1	0.991±0.136	0.9137±0.128	0.691±0.172	1.072±0.057	1.989±0.154	0.935±0.045
SYNV1	0.866±0.081	1.032±0.130	0.678±0.132	0.7486±0.096	0.748±0.096	0.640±0.118
CANX	0.817±0.113	1.594±0.320	0.888±0.071	1.198±0.047	3.502±0.147	1.066±0.111
EDEM1	0.754±0.123	0.8021±0.096	0.920±0.143	0.890±0.126	1.544±0.249	0.889±0.038
EDEM2	0.713±0.116	0.802±0.096	0.720±0.107	0.804±0.099	1.544±0.249	1.303±0.094
BIP	0.746±0.088	0.691±0.104	0.857±0.121	1.236±0.085	1.428±0.057	1.112±0.164
NOXA	0.996±0.137	0.7291±0.173	1.045±0.288	0.703±0.155	2.362±0.307	1.266±0.156

Western Blot analysis

	WT	Ter349Glu	Ter349Glu/WT
**Bip**	0.6270 ± 0.165	0.8227 ± 0.095	1.312
**CHOP**	0.222 ± 0.092	0.675 ± 0.097	3.040
**pATF6**	0.461 ± 0.108	0.603 ± 0.014	1.308
**IL-1b**	0.6693 ± 0.059	1.291 ± 0.118	1.929
