# Supplementary material for: An activated unfolded protein response promotes retinal degeneration and triggers an inflammatory response in the mouse retina
Source: Cell Death Dis. 2014 Dec 18;5(12):e1578–. doi: 10.1038/cddis.2014.539 (PMC4454166; doi:10.1038/cddis.2014.539)
Supplement: Supplementary Table 5 [file cddis2014539x9.pdf]

Table S5. Microglia is activated in ADRP retina: Immunohistochemical analysis .

Number of positive cells	C57BL/6-p15	C57BL/6-p30	T17MRHO+/-:p15	T17MRHO+/-:p30	T17M RHO/C57Bl6-p15	T17M RHO/C57Bl6-p30
F4/80	11.56 ± 0.4444	12.56 ± 0.1111	19.33 ± 0.6939	15.89 ± 0.6759	1.67	1.26
Iba1	20.00 ± 1.018	15.44 ± 0.7778	27.78 ± 1.544	25.78 ± 1.128	1.4	1.7

Western Blot analysis:

	C57BL6	Ter349Glu	Ter349Glu/C57BL6
IBA1	0.047 ± 0.003	0.091 ± 0.010	1.9
