# Supplementary material for: An activated unfolded protein response promotes retinal degeneration and triggers an inflammatory response in the mouse retina
Source: Cell Death Dis. 2014 Dec 18;5(12):e1578–. doi: 10.1038/cddis.2014.539 (PMC4454166; doi:10.1038/cddis.2014.539)
Supplement: Supplementary Table 6 [file cddis2014539x10.pdf]

Table S6. The IL-1b injection leads to retinal degeneration in the wild retina as measured by ERG, OCT and histological analyses.

ERG

Scotopic ERG	PBS	IL-1β	IL-1b/PBS
A-wave	243.5 ± 8.549	198.4 ± 8.522	0.81
B-wave	532.5 ± 15.91	448.3 ± 11.74	0.84

SD-OCT

Retina	PBS	IL-1b	Il-1b/PBS
Superior	58.43 ± 0.854	52.49 ± 1.214	0.89
Inferior	57.72 ± 0.711	51.12 ± 0.588	0.88

H@E staining. Number of photoreceptor rows in the ONL

PBS	IL-1b	Il-1b/PBS
11.80± 0.711	8.400± 0.509	0.71
